# Wenxin Keli Regulates Mitochondrial Oxidative Stress and Homeostasis and Improves Atrial Remodeling in Diabetic Rats

**DOI:** 10.1155/2020/2468031

**Published:** 2020-02-13

**Authors:** Mengqi Gong, Ming Yuan, Lei Meng, Zhiwei Zhang, Gary Tse, Yungang Zhao, Yue Zhang, Meng Yuan, Xue Liang, Guanwei Fan, Gan-Xin Yan, Guangping Li, Tong Liu

**Affiliations:** ^1^Tianjin Key Laboratory of Ionic-Molecular Function of Cardiovascular Disease, Department of Cardiology, Tianjin Institute of Cardiology, Second Hospital of Tianjin Medical University, Tianjin 300211, China; ^2^Tianjin Key Laboratory of Exercise Physiology and Sports Medicine, Department of Health & Exercise Science, Tianjin University of Sport, Tianjin 300381, China; ^3^First Teaching Hospital of Tianjin University of Traditional Chinese Medicine, Tianjin 300193, China; ^4^Tianjin Key Laboratory of Translational Research of TCM Prescription and Syndrome, Tianjin, China; ^5^Lankenau Institute for Medical Research and Lankenau Medical Center, Wynnewood, PA, USA; ^6^Fuwai Huazhong Cardiovascular Hospital, Zhengzhou, China

## Abstract

Mitochondrial dysfunction and oxidative stress play an important role in the pathogenesis of both atrial fibrillation (AF) and diabetes mellitus (DM). Wenxin Keli (WXKL), an antiarrhythmic traditional Chinese medicine, has been shown to prevent cardiac arrhythmias through modulation of cardiac ion channels. This study tested the hypothesis that WXKL can improve atrial remodeling in diabetic rats by restoring mitochondrial function. Primary atrial fibroblasts of neonatal SD rats were divided into four groups: control, hydrogen peroxide (H_2_O_2_), H_2_O_2_+WXKL 1 g/L, and H_2_O_2_+WXKL 3 g/L groups. Intracellular mitochondrial membrane potential (MMP), reactive oxygen species (ROS), and mitochondrial oxygen consumption were measured. SD male rats were randomly divided into three groups: control, DM, and DM+WXKL groups. Rats in the DM+WXKL group were treated with daily gavage of WXKL at 3 g/kg. After eight weeks, echocardiography, hemodynamic examination, histology, electrophysiology study, mitochondrial respiratory function, and western blots were assessed. H_2_O_2_ treatment led to increased ROS and decreased intracellular MMP and mitochondrial oxygen consumption in primary atrial fibroblasts. WXKL improved the above changes. DM rats showed increased atrial fibrosis, greater left atrial diameter, lower atrial conduction velocity, higher conduction heterogeneity, higher AF inducibility, and lower mitochondrial protein expression, and all these abnormal changes except for left atrial diameter were improved in the DM+WXKL group. WXKL improves atrial remodeling by regulating mitochondrial function and homeostasis and reducing mitochondrial ROS in diabetic rats.

## 1. Introduction

Atrial fibrillation (AF) is the most common arrhythmia observed in the clinic. In recent years, its prevalence has increased with the aging of the population. AF is a risk factor for thromboembolism, which increases the risk of stroke and all-cause mortality [1, 2]. The development of AF is characterized by structural, electrical, and metabolic remodeling of the atria [3]. Previous studies have demonstrated that the development of AF is related to a variety of risk factors, but the pathogenesis of AF is complex and remains incompletely understood.

Diabetes mellitus (DM) is an independent risk factor for AF [4, 5]. Our previous work and those of other groups have demonstrated the importance of mitochondrial dysfunction and oxidative stress in mediating atrial remodeling in DM, which is associated with higher AF incidence [6]. Mitochondrial dysfunction is associated with the excessive release of reactive oxygen species (ROS) and cytochrome c, which initiates apoptosis. Abnormal biomarkers of oxidative stress have been found in AF patients and animal models of AF [7]. For example, the oxidative ratio of serum glutathione and cysteine in AF patients was significantly elevated. Prooxidative genes were upregulated, and antioxidant genes were downregulated in atrial appendages of AF patients [8, 9]. Montaigne et al. showed that mitochondrial dysfunction of atrial muscle before cardiac surgery was associated with the development of postoperative AF [10]. Currently, antioxidant drugs have been a potential therapeutic strategy of AF. Mitochondria play a vital role in myocardial energy metabolism and redox equilibrium. Energy metabolism disorder affects myocardial excitability and contraction. Therefore, cardiac mitochondria may be a specific therapeutic target.

Wenxin Keli (WXKL) is a traditional Chinese medicine, which is composed of Codonopsis, Rhizoma Polygonati, Notoginseng, Amber, and Nardostachys [11]. In Chinese medicine clinics, WXKL is used for the treatment of different cardiac arrhythmias, including AF. The antiarrhythmic effects of WXKL have been attributed to shortening of the action potential duration with simultaneous prolongation of the atrial effective refractory period. This leads to postrepolarization refractoriness, which can effectively prevent AF. WXKL exerts atrial-selective inhibition of peak Na current (*I*_Na_) and thus effectively suppresses AF in experimental models of AF [12–14]. Our previous clinical study demonstrated that WXKL combined with amiodarone markedly shortened conversion time with no adverse reactions or proarrhythmic effects for AF patients [15]. WXKL was also used with other antiarrhythmic drugs in the clinic to prevent the perpetuation of AF [16].

Hydrogen peroxide (H_2_O_2_) as a potent oxidant induces mitochondrial dysfunction by increasing ROS production, which leads to reduced antioxidant activity and induced mitochondrial apoptotic signaling [17, 18]. High-fat diet- (HFD-) and low-dose streptozotocin- (STZ-) treated rats provide an animal model for type 2 DM (T2DM) [19]. The HFD+STZ model, which progresses from insulin resistance to hypoinsulinemia and hyperglycaemia, mimics the natural T2DM pathogenesis in humans and is suitable to investigate the pathogenesis of diabetic complications [19]. In this study, we aim to investigate the H_2_O_2_-induced oxidative stress in vitro and the atrial remodeling, atrial muscle mitochondrial function, and atrial electrophysiological properties in HFD/STZ-induced type 2 diabetic rats and the potential therapeutic effects of WXKL.

## 2. Materials and Methods

### 2.1. Isolation and Culturing of Fibroblasts

Primary cultures of atrial fibroblasts were isolated from 3-day-old neonatal Sprague-Dawley (SD) rats. In brief, neonatal SD rats were sacrificed by cervical dislocation, then sterilized in 75% ethanol, and subjected to subsequent operations in a clean bench. The left atrium was separated from the exposed heart; then, it was cut to small pieces of 1 mm^3^ and put in phosphate-buffered saline (PBS) solution without Ca^2+^ and Mg^2+^. Atrial tissues were repeat digested by trypsin type II collagenase buffer (0.125%, respectively) until the tissue completely dissipated. All the cell suspensions were centrifuged for 5 min at 1000 r/min. Isolated cells were resuspended in Dulbecco's modified Eagle's medium (DMEM), supplemented with 10% fetal bovine serum (FBS, Gibco) and 100 IU/mL penicillin-streptomycin (Gibco), and plated on 10 cm culture dishes for 2 h until the fibroblasts adhered to the dishes in a humidified atmosphere at 37°C under 5% CO_2_. The preseeding medium containing cardiomyocytes was removed, and the culture medium was added to continue to culture the atrial fibroblasts. The culture medium was changed every 48 hours. Atrial fibroblasts were used in subsequent experiments and incubated in FBS free medium for 24 h before treatment. The cells were pretreated with vehicle or WXKL (1 g/L or 3 g/L) for 1 hour and then stimulated with 300 *μ*M hydrogen peroxide (H_2_O_2_) for 24 hours. The cells were randomly divided into the following groups: control, H_2_O_2_, H_2_O_2_+WXKL 1 g/L, and H_2_O_2_+WXKL 3 g/L groups.

### 2.2. Experimental Animals and Protocol

SD male rats aged 8 weeks (average body weight of 200 ± 20 g) were purchased from Beijing HuaFuKang Bioscience Co., Ltd. (Beijing, China). These rats were fed under controlled conditions with a constant temperature of 25 ± 2°C, a humidity of 60 ± 5%, and a 12 h day/night cycle. All rats accepted normal chow diet of 1 week for acclimation before initiation of the study. All rats were assigned randomly to the following 3 groups by a random number table: control, DM, and DM+WXKL groups. These rats of a diabetic group, including DM and DM+WXKL groups, were fed with HFD (60 kcal% fat, 20 kcal% carbohydrate, and 20 kcal% protein; H10060, Beijing HuaFuKang Bioscience Co., Ltd., Beijing, China), while the control group was fed with normal chow diet throughout the whole study. After 4-week feeding, all animals were overnight fasted; then, the diabetic group was induced by a single tail vein injection of STZ (30 mg/kg; Sigma-Aldrich, St. Louis, MO, USA) in 0.1 M citrate buffer (pH 4.5). The rats in the control group were injected with the citrate buffer vehicle. 72 hours after injection, fasting blood glucose levels (FBG) were measured, and rats in the STZ injection group with blood glucose levels > 11 mmol/L were considered diabetic models and would be used in future experiments. This model involves a combination of a diet high in fat to bring about hyperinsulinemia, insulin resistance, and/or glucose intolerance followed by treatment with the *β*-cell toxin STZ, which results in a severe reduction in functional *β*-cell mass [19–21]. WXKL was kindly supplied by Shandong Buchang Pharmaceutical Co., Ltd. (Heze, China). The DM+WXKL group was given to the rats by daily lavage of WXKL (3 g/kg) dissolved in distilled water, and the other groups daily received distilled water only. We performed the following experiments on all rats after 8 weeks of treatment. Animal protocols adopted in the present study were approved by the Experimental Animal Administration Committee of Tianjin Medical University (Approval No. TMUaMEC2019004), which follows the guidelines established by the U.S. National Institutes of Health.

### 2.3. Mitochondrial Membrane Potential and ROS Measurement of Atrial Fibroblasts

The atrial fibroblasts were incubated with JC-1 staining buffer in the dark for 20 min at 37°C and washed twice with PBS without Ca^2+^ and Mg^2+^. The processes of measurement were used with Olympus FV1000 LSCM under excitation wavelength of 488 nm and 543 nm. The decreased relative fluorescence proportion of aggregate JC-1 (red)/monomeric JC-1 (green) indicates a decrease in the mitochondrial membrane potential (MMP). ROS were detected using the 2′,7′-dichlorofluorescin diacetate (DCFH-DA, Sigma-Aldrich) fluorescent probe. The cells were incubated with 10 *μ*M DCFH-DA in PBS without Ca^2+^ and Mg^2+^ in the dark for 20 min at 37°C. Fluorescence was determined at 488 nm for excitation and 525 nm for emission. Microscope images were saved as TIFF files and processed for densitometric quantification with ImageJ version 1.46 (NIH). Software settings were kept the same for every image analyzed.

### 2.4. Assay of Cellar Mitochondrial Oxygen Consumption Rate

The mitochondrial oxygen consumption rate (OCR) of atrial fibroblasts was measured using the Seahorse Bioscience XF24 Extracellular Flux Analyzer (Seahorse Bioscience), and this process was according to instructions of the manufacturer and Lay et al. [22]. Briefly, atrial fibroblasts were cultured on a Seahorse XF microplate as required with an equal number per well and treated according to the protocol. The Seahorse XF24 extracellular flux assay plate was hydrated for 18 hours without CO_2_ in XF calibration buffer at 37°C before assay. 10 *μ*M oligomycin, 10 *μ*M carbonyl cyanide 4-(trifluoromethoxy) phenylhydrazone (FCCP), and 10 *μ*M antimycin A (AA)+retenone were injected in turn during the process of assay.

### 2.5. Echocardiographic Assessments

After 8 weeks of treatment in each group, rats were allowed to inhale isoflurane (1.5 L/min) for anesthesia and placed on a 37°C thermal table in the supine position to measure transthoracic echocardiography. Echocardiographic parameters, including left atrial (LA) diameter (LAD), left ventricular posterior wall thickness (LVPWT), interventricular septal thickness (IVST), left ventricular end-diastolic dimension (LVEDD), and left ventricular end-systolic dimension (LVESD), were obtained in the parasternal long-axis view using a specified small animal ultrasound system (VisualSonics Vevo 2100, SONICS, Newtown, CT, USA) by a senior blinded operator. These parameters were measured by 2-dimensional imaging during 3 consecutive cardiac ejection cycles. Left ventricular ejection fraction (LVEF) was acquired in line with ventricular volumes calculated by the Teichholz formula:

(1) $$\begin{array}{c} \text{Left ventricular end}‐\text{diastolic volume }\left(\text{LVEDV}\right)=\frac{7}{2.4+\text{LVEDD}}*{\text{LVEDD}}^{3},\\ \text{Left ventricular end}‐\text{systolic volume }\left(\text{LVESV}\right)=\frac{7}{2.4+\text{LVESD}}*{\text{LVEDD}}^{3},\\ \text{LVEF}=\frac{\text{LVEDV}-\text{LVESV}}{\text{LVEDV}}*100\%. \end{array}$$

### 2.6. Surface Electrocardiographic and Hemodynamic Experiment and Sample Collection

At the end of echocardiographic examination, each rat underwent right carotid artery cannulation allowing measurements of hemodynamic parameters using a Millar catheter during electrocardiographic (ECG) monitoring. Aortic systolic blood pressure (SBP), diastolic blood pressure (DBP), and mean blood pressure (MBP) were recorded carefully after a stabilization period. Subsequently, the cannula was inserted through the aortic valve to the left ventricle to record the left ventricular end-diastolic pressure (LVEDP) and maximum increasing or decreasing rate of left ventricular pressure (±dp/dt max). 10 stable waveforms of electrocardiogram were selected to measure heart rate, P wave duration, PR interval, QRS duration, and QT interval. Finally, rats were instantly euthanized. Then, blood samples were obtained from the right ventricle and LA tissues were rapidly separated. Samples for histological assessment were soaked in neutral buffered 10% formalin solution, and samples for molecular biological experiments were rapidly frozen in liquid nitrogen and stored at -80°C.

### 2.7. Serum Biochemical and Biomarker Measurements

FBG levels were detected with the blood glucose meter and test strips each week. Serum biochemical parameters including total cholesterol (TC), triglycerides (TG), low-density lipoprotein cholesterol (LDL-C), high-density lipoprotein cholesterol (HDL-C), and creatinine (Cr) level were detected using a fully automatic biochemical analyzer. Rat ELISA kits were used to detect the serum levels of insulin and high-sensitivity C-reactive protein (hs-CRP) (Wuhan Huamei Biological Engineering Co, Ltd., China); also, lipid peroxidation malondialdehyde (MDA) and superoxide dismutase (SOD) levels were measured according to instructions of the manufacturer (Nanjing Jiancheng Bioengineering Institute, China).

### 2.8. Electrophysiological Study

The rats were intraperitoneally injected with 3% pentobarbital sodium (50 mg/kg) for anesthesia. The heart was quickly isolated and placed in 4°C Tyrode's solution to wash out residual blood and to remove residual lung tissue. Then, the aorta was cannulated and connected to a Langendorff perfusion system. The Langendorff perfusion system was filled with 37°C Tyrode's solution equilibrated with 5% CO_2_ and 95% O_2_. Preperfusion was performed for 20 minutes to minimize endogenous release of catecholamine residual effects. Some hearts could not maintain normal rhythms after 20 minutes and were abandoned.

Epicardial activating electrical mapping was recorded by a 6 × 6 electrode microelectrode (electrode impedance: 1.5-1.7Q, PA03606060101, multielectrode probe array) on the epicardial surface of the left and right atrium. Data were recorded by multichannel systems (EMS64-USB-1003, United Kingdom). Activation waveforms acquired were amplified by a filter amplifier and thereby transmitted to the connected computer. All activation time was digitized and then used to draw activation maps. The activation times were calculated as the point of maximal negative slope of activation waveforms. Conduction velocity (CV), inhomogeneity index, and absolute inhomogeneity were calculated by EMapScope 4.0 software (MappingLab Ltd., UK).

Three silver bipolar electrodes were placed on the left atrium, right atrium, and right ventricular apex after recorded epical mapping, and the entire recorded process was connected to a custom-made computer software program (Electrophysiological Recording System, TOP-2001, HTONG Company, Shanghai, China). There were 3 basic cycle lengths (BCLs) of basic stimuli (S1), including 200 ms, 150 ms, and 100 ms. When the sinus cycle length (SCL) was shorter than 200 ms, we used the SCL subtracted by 10 ms to replace BCL of 200 ms. The interatrial conduction time (IACT) was measured during right atrium pacing. Then, atrial-ventricular Wenckebach cycle length conduction (AVWCL) was measured by right atrial (RA) incremental pacing. The S1S1 interval was decreased in 5 ms steps until ventricular rhythm was not 1 : 1 following atrial pacing. 8 S1 were followed by a premature extra stimulus (S2) to measure the atrial effective refractory period (ERP). The S1S2 interval was decreased in 2 ms steps until the longest S1S2 interval that failed to capture the depolarization. The stimulation order of burst pacing was from the right atrium to the left atrium. AF induction was verified by burst pacing (cycle lengths of 50 ms, 40 ms, and 30 ms) for 3 seconds, respectively, which was performed 5 times with 30-second intervals. AF was defined as rapid, irregular atrial response longer than 1 second. AF inducibility was defined as the percentage of burst pacing leading to AF episodes.

### 2.9. Isolation of Atrial Mitochondria and Measurement of Atrial MMP

Rats were euthanized with 3% pentobarbital sodium (50 mg/kg), and median sternotomy was immediately performed. The LA mitochondria were isolated, and the mitochondrial protein content was assayed according to our previous studies. Atrial MMP was assessed by JC-1 staining (Beyotime, Jiangsu, China). 300 *μ*g of mitochondrial protein in 2 mL of respiration medium were incubated with JC-1 working solution at 37°C in the dark for 10 min.

### 2.10. Western Blot Analysis

Frozen LA tissues were homogenized in liquid nitrogen, and the total protein was extracted by RIPA lysis buffer. The lysates were centrifuged at 15,000g for 20 minutes, and the supernatants were collected. Protein concentrations were measured by the bicinchoninic acid (BCA) protein assay regent kit (Thermo Scientific, USA). All sample proteins loaded equally with 60 *μ*g were separated by sodium dodecyl sulfate-polyacrylamide gel electrophoresis (SDS-PAGE) and transferred by electrophoresis to a polyvinylidene difluoride (PVDF) membrane. Subsequently, PVDF membranes were blocked for 1 h with 5% nonfat dry milk or bovine serum albumin (BSA) in Tris-buffered saline containing 0.1% Tween 20 (TBST), then incubated at 4°C overnight with a primary antibody. Then, the membrane was washed with TBST and incubated with the horseradish peroxidase-conjugated secondary antibody for 1 hour. The primary antibodies used were as follows: *β*-actin (1 : 5000, 60008-1-Ig, Proteintech), mitochondrial transcription factor A (TFAM, 1 : 2000, ab131607, Abcam), dynamin-related protein (Drp1, 1 : 1000, ab5678, Abcam), mitofusin 2 (Mfn2, 1 : 1000, ab56889, Abcam), transforming growth factor-*β* (TGF-*β*, 1 : 1000, ab190503, Abcam), nuclear factor kappa-b (NF-*κ*b, 1 : 1000, ab90532, Abcam), Bcl-2 (1 : 1000, ab59348, Abcam), Bax (1 : 5000, ab32503, Abcam), collagen I (1 : 1000, ab34710, Abcam), collagen III (1 : 5000, ab7778, Abcam), and *α*-smooth muscle actin (*α*-SMA, 1 : 5000, ab5694, Abcam). *β*-Actin was evaluated as a loading control. The reactions were visualized using Tanon 5200 Multi Chemiluminescent Imaging System (Tanon Science & Technology Co., Ltd., Shanghai, China).

### 2.11. Histological Studies

The tissues were cut into 5 *μ*m cross sections, then stained with hematoxylin and eosin (HE) to observe the morphological changes and Masson's trichrome stain to evaluate interstitial fibrosis. Digital images were scanned by Image-Pro Plus 6.0, and the extent of fibrosis was determined by dividing the area of collagen deposition by the entire cardiac tissue area. Five randomly selected sections of each group were utilized for quantification. The cardiac collagen volume fraction and cross-sectional area were analyzed using SPSS 17.0.

### 2.12. Statistical Analysis

Data were presented as mean ± standard deviation (SD). Differences between the groups were analyzed for statistical significance using the one-way analysis of variance (ANOVA) followed by the Least Significant Difference (LSD) test. All data were analyzed using SPSS 17.0, and *P* values < 0.05 were considered significantly altered.

## 3. Results

### 3.1. WXKL Decreases ROS and Improves Mitochondrial Function of Atrial Fibroblasts

**Figure 1:**
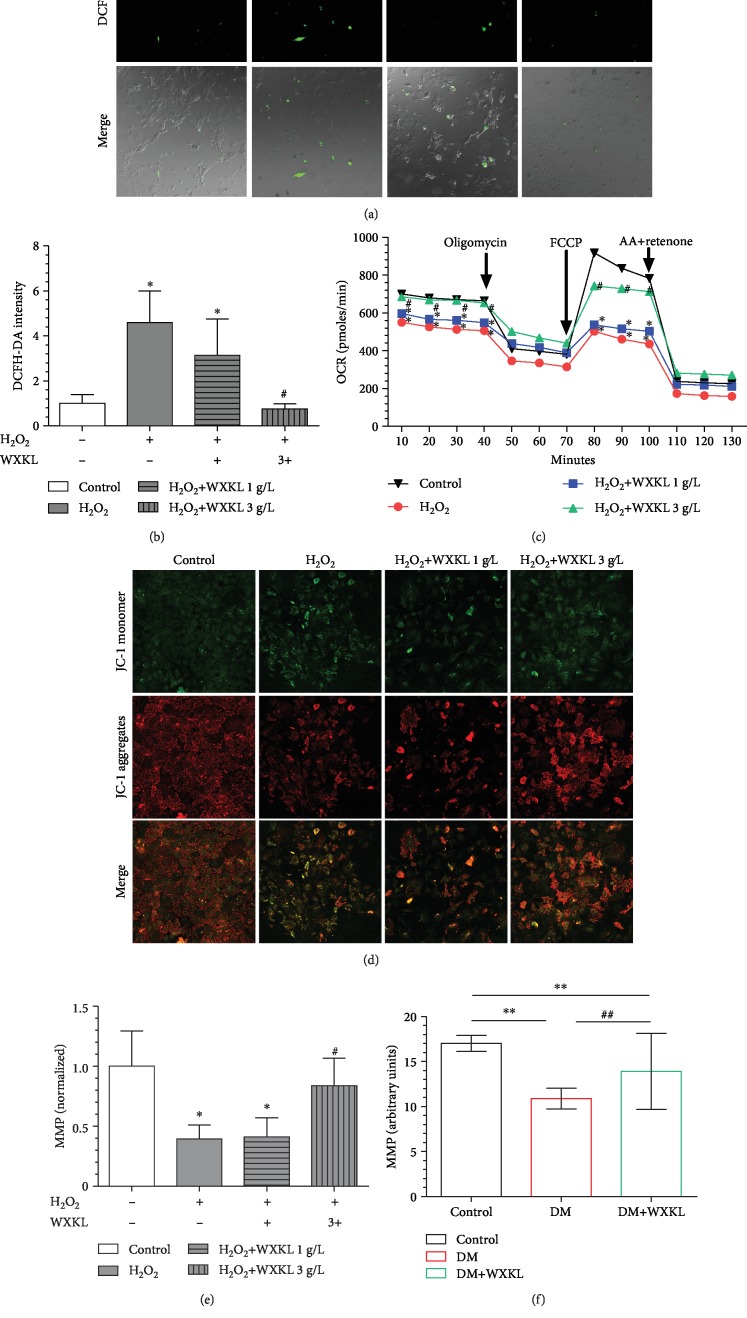
WXKL inhibited H_2_O_2_-induced oxidative stress and mitochondrial dysfunction. (a) Representative confocal microscopy images of atrial fibroblasts that were stained with DCFH-DA and merged with cells. (b) Quantification of ROS by DCFH-DA intensity. (c) Analysis results of OCR from Seahorse XF24 Extracellular Flux Analyzer. Oligomycin inhibits ATP synthase (complex V), FCCP uncouples oxygen consumption from ATP production, and AA+retenone inhibits complexes I and III, respectively. Sequential compound injections measure basal respiration, ATP production, proton leak, maximal respiration, spare respiratory capacity, and nonmitochondrial respiration. The decrease in OCR upon injection of oligomycin represents the portion of basal respiration that was being used to drive ATP production. The maximal OCR attained by adding the uncoupler FCCP. Nonmitochondrial respiration due to a subset of cellular enzymes that continue to consume oxygen after AA+retenone addition. (d) Representative confocal microscopy images of atrial fibroblasts that were stained with JC-1 dye. (e) Quantification of MMP of atrial fibroblasts by JC-1 aggregates/JC-1 monomer. (f) Quantification of MMP from LA of rats between the 3 groups. WXKL: Wenxin Keli; H_2_O_2_: hydrogen peroxide; DCFH-DA: 2′,7′-dichlorofluorescin diacetate; ROS: reactive oxygen species; OCR: oxygen consumption rate; MMP: mitochondrial membrane potential; AA: antimycin A; FCCP: carbonyl cyanide 4-(trifluoromethoxy) phenylhydrazone. Values are presented as mean ± SD. ^∗^Compared with control, *P* < 0.05. ^#^Compared with H_2_O_2_, *P* < 0.05; *n* = 3 independent experiments. ^∗∗^Compared with the control group of rats, *P* < 0.05. ^##^Compared with the DM group of rats, *P* < 0.05. *n* = 5 per group.

DCFH-DA fluorescence was measured in atrial fibroblasts from each experimental group (Figure 1(a)). ROS was significantly higher in the H_2_O_2_ group compared with the control group (4.59 ± 1.41 vs. 1 ± 0.39, *P* < 0.01, Figure 1(b)). The concentration of WXKL used in our previous clinical study was doubled, which was used in clinical practice. In this in vitro cell experiment, we compared doubled concentration (1 g/L) and six-fold concentration (3 g/L) of WXKL. The ROS level in the H_2_O_2_+WXKL 1 g/L group and the H_2_O_2_+WXKL 3 g/L group was 3.14 ± 1.61 and 0.75 ± 0.22, respectively (Figure 1(b)). The ROS was markedly decreased in the H_2_O_2_+WXKL 3 g/L group (*P* < 0.01), while ROS already had a decreased trend in the H_2_O_2_+WXKL 1 g/L group (*P* = 0.053) compared with the H_2_O_2_ group.

OCR measurements then permitted us to investigate whether WXKL improved mitochondrial function. Basal respiration and maximal respiration were also decreased in the H_2_O_2_ group compared with the control group, and the above changes were improved in the H_2_O_2_+WXKL 3 g/L group (Figure 1(c)). There were no differences of ATP production and nonmitochondrial respiration in all groups (Figure 1(c)). MMP was quantified using the JC-1 dye (Figure 1(d)). The MMP was significantly reduced in the H_2_O_2_ group (0.39 ± 0.12, *P* < 0.01). The MMP was similar between the H_2_O_2_ group and the H_2_O_2_+WXKL 1 g/L group (Figure 1(e)). And the MMP was improved in H_2_O_2_+WXKL 3 g/L.

### 3.2. WXKL Improves Left Atrial MMP in Type 2 Diabetic Rats

We successfully established a rat model of type 2 diabetes by high-fat feeding+low STZ injection. FBG was stabilized in a hyperglycemic state within 8 weeks of the whole study process. The general characteristics of the 3 groups are presented in Table 1. Thus, after four weeks of high-fat diet, FBG was slightly elevated in the DM group (8.79 ± 0.56 mmol/L, *P* < 0.01) and DM+WXKL group (8.41 ± 0.85 mmol/L, *P* < 0.01) compared with the control group (7.12 ± 0.61 mmol/L), and there was no difference in FBG between the two diabetic groups (*P* = 0.28). Blood glucose was significantly higher in the DM (25.76 ± 5.26 mmol/L, *P* < 0.01) and DM+WXKL groups (25.51 ± 4.93 mmol/L, *P* < 0.01) compared with the control group (5.98 ± 0.93 mmol/L). Serum insulin level, quantified by ELISA, was significantly higher in the DM (32.65 ± 8.69 pmol/L) and DM+WXKL groups (31.32 ± 7.25 pmol/L) when compared to the control group (20.44 ± 5.71 pmol/L).

After 8 weeks of WXKL administration, mitochondria of the left atrium were extracted for MMP detection (Figure 1(f)). MMP was decreased in the DM group compared with the control group (10.90 ± 1.14 vs. 17.02 ± 0.89, *P* < 0.01). The MMP of the DM+WXKL group was also lower than that of the control group (13.92 ± 4.21 vs. 17.02 ± 0.89, *P* = 0.02). However, the MMP of the DM+WXKL group was improved compared with that of the DM group (13.92 ± 4.21 vs. 10.90 ± 1.14, *P* = 0.02).

### 3.3. WXKL Improves Atrial Remodeling and Prevents Atrial Fibrillation

**Figure 2:**
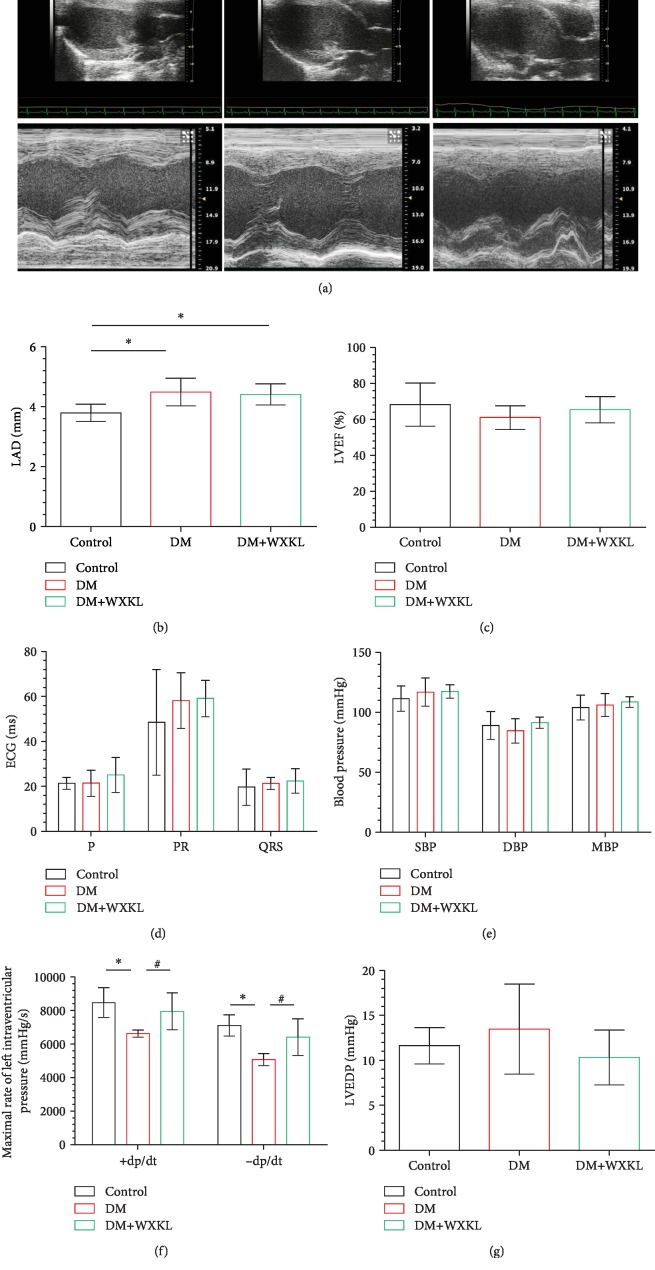
Effect of WXKL on echocardiographic, electrographic, and hemodynamic data in diabetic rats. (a) Representative 2-dimensional and M-mode of echocardiographic images. Analysis results of LAD (b) and LVEF (c). (d) Quantification of ECG parameters (P: P wave duration; PR: PR interval; QRS: QRS duration). (e–g) Results of hemodynamic studies. SBP: systolic blood pressure; DBP: diastolic blood pressure; MBP: mean blood pressure; +dp/dt: maximal increasing rate of left intraventricular pressure; -dp/dt: maximal decreasing rate of left intraventricular pressure; LVEDP: left ventricular end-diastolic pressure; LAD: left atrial diameter; LVEF: left ventricular ejection fraction; ECG: electrocardiogram. Values are presented as mean ± SD. ^∗^Compared with the control group, *P* < 0.05. ^#^Compared with the DM group, *P* < 0.05; *n* = 8 per group.

Representative echocardiographic images of the three groups are shown in Figure 2(a). We found that LAD increased in the DM group compared with the control group (4.49 ± 0.46 mm vs. 3.79 ± 0.29 mm, *P* = 0.001, Figure 2(b)). LAD in the DM+WXKL and DM groups were not significantly different (4.41 ± 0.35 vs. 4.49 ± 0.46, *P* = 0.691). There were no differences in LVEF, IVS, and LVPW between the three groups (Figure 2(c) and Table 1). For ECG data, P wave duration, PR interval, and QRS duration were not significantly different between the three experimental groups (Figure 2(d)).

We measured hemodynamics through a Miller pressure catheter. SBP, DBP, and MBP were not significantly different between the three groups (Figure 2(e)). However, +dp/dt and -dp/dt in the DM group were significantly lower than those in the control group (6625.83 ± 209.75 vs. 8469.54 ± 882.15 (*P* < 0.01) and 5069.63 ± 355.39 vs. 7113.05 ± 632.80 (*P* < 0.01), Figure 2(f)). The above parameters were improved in the DM+WXKL group. There was no difference of LVEDP between the three groups (Figure 2(g)).

**Figure 3:**
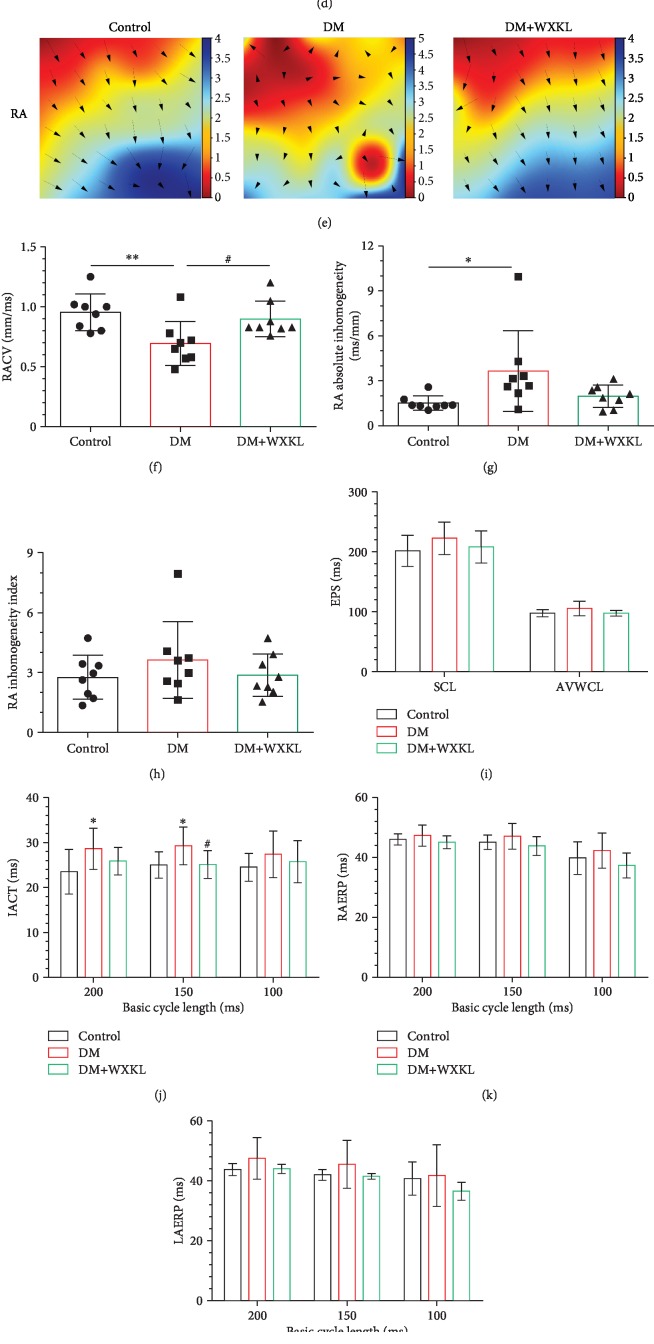
Effect of WXKL on epicardial electrical mapping and AF incidence in diabetic rats. Representative epicardial electrical mapping recorded of LA (a) or RA (e). Conduction velocity (CV) of LA (b) or RA (f), absolute inhomogeneity of LA (c) or RA (g), and inhomogeneity index of LA (d) or RA (h). (i) Parameters from EPS (SCL: sinus cycle length; AVWCL: atrioventricular Wenckebach cycle length). (j–l) Quantifications of interatrial conduction time (IACT), right atrium effective refractory period (RAERP), and left atrium effective refractory period (LAERP) at basic cycle lengths of 200, 150, and 100 ms. (m) Representative AF episodes induced by LA burst pacing. (n) Quantification of the inducibility of AF. Values are presented as mean ± SD. ^∗^Compared with the control group, *P* < 0.05. ^∗∗^Compared with the control group, *P* < 0.01. ^#^Compared with the DM group, *P* < 0.05; *n* = 8 per group.

Electrophysiological parameters were obtained from Langendorff-perfused hearts. We firstly recorded electrical conduction mapping of LA (Figure 3(a)) and RA (Figure 3(e)). Mapping images showed that left atrial conduction velocity (LACV) was significantly lower in the DM group than in the control group (0.66 ± 0.29 mm/ms vs. 1.10 ± 0.30 mm/ms, *P* = 0.01, Figure 3(b)). This was significantly improved in the DM+WXKL group (1.02 ± 0.33 mm/ms vs. 0.66 ± 0.29 mm/ms, *P* = 0.03, Figure 3(b)). LA conduction dispersion was higher in the DM group than in the control group (absolute 3.81 ± 2.63 vs. 1.39 ± 0.43*P* < 0.01, index 3.07 ± 1.80 vs. 1.87 ± 0.59, *P* = 0.046; Figures 3(c) and 3(d)). LA conduction dispersion of the DM+WXKL group was reduced compared with that of the DM group. LA electrical conductions of the control group were uniform and showed an orderly spread to the surrounding tissue. However, LA conduction of the DM group was disordered and there were abnormal conduction positions in the wave conduction, but it was restored in the DM+WXKL group. Comparable results were observed in the mapping images of RA electrical conduction maps (Figures 3(e) and 3(f)–3(h)).

There were no significant differences of SCL and AVWCL between the three groups (Figure 3(i)). The IACT of the DM group was slightly longer than that of the control group in 200 ms and 150 ms of BCL (Figure 3(j)). We also measured RAERP and LAERP in all three BCLs (Figures 3(k) and 3(l)). RAERP and LAERP in the three groups were also statistically indistinguishable. A representative AF episode induced by LA burst pacing is shown in Figure 3(m). We observed that the AF incidence was significantly higher in the DM group than in the control group (50.94% vs. 18.12%, *P* < 0.01, Figure 3(n)). The AF incidence in the DM+WXKL group was considerably lower than that in the DM group (19.06% vs. 50.94%, *P* < 0.01, Figure 3(n)). Meanwhile, there was no significant difference between the DM+WXKL group and the control group (*P* = 0.93).

**Figure 4:**
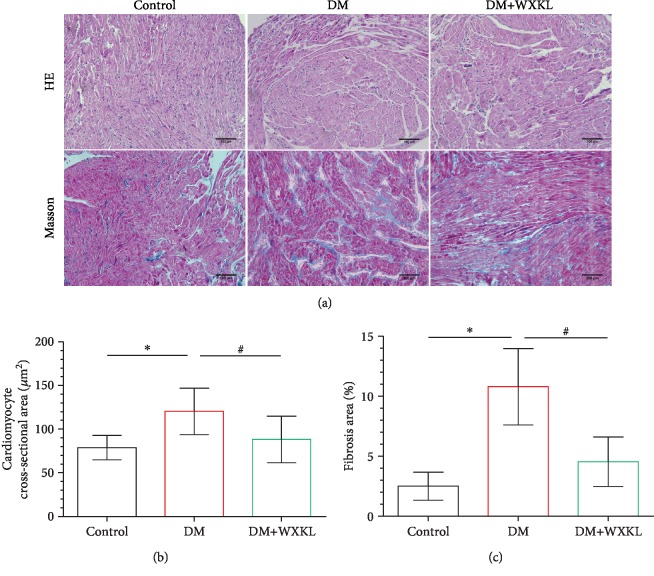
WXKL improves the atrial modeling in diabetic rats. (a) Representative images of right atrial HE and Masson's trichrome staining (200x). (b) Cardiomyocyte cross-sectional area and (c) interstitial fibrosis. Values are presented as mean ± SD. ^∗^Compared with the control group, *P* < 0.05. ^#^Compared with the DM group, *P* < 0.05; *n* = 8 per group.

H&E and Masson's trichrome staining was used to detect morphological changes in the RA (Figure 4(a)). Cardiomyocytes in the control group had normal morphology, but its cross-sectional area was higher in the DM group (Figure 4(b)). The ratio of myocardial interstitial fibrosis was higher in the DM group compared with the control group (10.78 ± 3.18% vs. 2.49 ± 1.18%, *P* < 0.001, Figure 4(c)). However, cardiomyocyte hypertrophy and fibrosis ratio were alleviated in the DM+WXKL group.

### 3.4. WXKL Improves Serum Oxidative Stress

Different serum biomarkers were measured. hs-CRP levels were higher in the DM group and DM+WXKL group than in the control group (Table 1). There was no difference between the DM group and the DM+WXKL group (17.66 ± 5.18 vs. 16.81 ± 3.65, *P* = 0.67). Lower SOD and higher MDA levels were detected in the DM group compared to the control group (Table 1). Serum levels of BUN, Cr, HDL-C, and LDL-C were not significantly different amongst the three groups (Table 1). Meanwhile, TC and TG were significantly higher in the DM group than in the control group, and these markers could not be aggravated by WXKL treatment (Table 1).

### 3.5. WXKL Upregulates Mitochondria-Associated Proteins and Reduces Apoptosis

**Figure 5:**
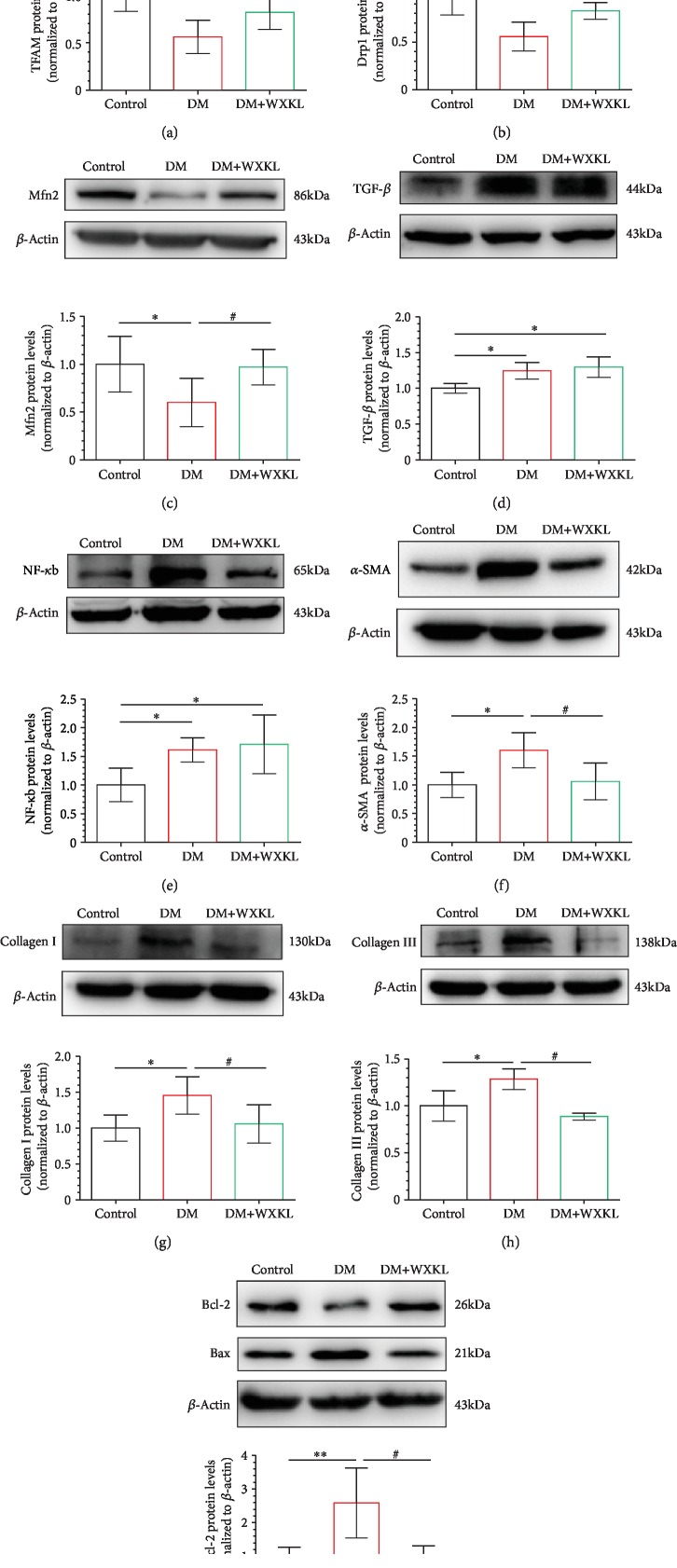
WXKL increases mitochondria-related proteins and improves fibrotic and apoptotic proteins. Representative western blot results and analysis of the protein expression in the three groups. (a–i) Mitochondrial transcription factor A (TFAM), dynamin-related protein 1 (Drp1), mitofusin 2 (Mfn2), transforming growth factor-*β* (TGF-*β*), nuclear factor kappa-b (NF-*κ*b), *α*-smooth muscle actin (*α*-SMA), collagen I, collagen III, and Bax/Bcl-2. Values are presented as mean ± SD. ^∗^Compared with the control group, *P* < 0.05. ^∗∗^Compared with the control group, *P* < 0.01. ^#^Compared with the DM group, *P* < 0.05; *n* = 5 independent experiments.

We firstly evaluated the expression of mitochondria-associated protein of LA tissue and found that the expression levels of TFAM, Drp1, and Mfn2 were significantly lower in the DM group compared with the control group (Figures 5(a)–5(c)). Encouragingly, expression levels of these mitochondria-associated proteins were significantly increased in the DM+WXKL group. We compared the expression levels of inflammatory proteins. TGF-*β* and NF-*κ*b were upregulated in the DM group (Figures 5(d) and 5(e)). However, the expression levels of two inflammatory proteins were not different between the DM+WXKL group and DM group. Compared with the control group, the expressions of fibrotic proteins, collagen I, collagen III, and *α*-SMA, were significantly increased in the DM group (Figures 5(f)–5(h)). These changes were significantly reversed in the DM+WXKL group.

Finally, we compared the apoptotic proteins between the three groups. It was found that the expression level of Bax was upregulated and the antiapoptotic protein Bcl-2 was downregulated in the DM group (Figure 5(i)). The expression levels of Bax and Bcl-2 were improved in the DM+WXKL group compared with the DM group and approached the expression level of the control group.

## 4. Discussion

In this study, we found that WXKL enhances mitochondrial function with improved basal and maximal respiration and reduces ROS production. WXKL treatment improved atrial remodeling and prevented AF by restoring mitochondrial function in diabetic rats.

DM is a well-recognized risk factor for the development of AF [4, 23–25]. The results of serum markers showed that there were abnormal changes in inflammation and oxidative stress levels in diabetic rats, which is consistent with our previous studies [26–28]. Mitochondrial dysfunction and molecular mechanisms underlying DM-related AF development have been reported [10, 24]. ROS is a critical factor mediating both DM and AF [5, 24, 29]. A variety of sources, including NADPH oxidase [26], mitochondrial enzymes [28], and xanthine oxidase [27], generate ROS in the context of AF [30, 31]. Mitochondria-derived ROS are considered the main ROS source of age-related AF [32]. Therefore, mitochondrial oxidative stress is a potential target [24].

Mitochondrial homeostasis is important for maintaining the normal function of mitochondria [33, 34]. Patients with T2DM and obesity demonstrated reduced expression of Mfn2, which may be related to the reduced function of mitochondria [35]. Excessive mitochondrial fission or slowing mitochondrial fusion leads to an increase in mitochondrial fragmentation [36]. A decreasing mitochondrial volume accelerates the speed of the electron transport chain. However, a reduced mitochondrial inner membrane area makes mitochondria susceptible to calcium overload, which initiates apoptosis [36]. Mfn2-deficient mice display enlarged cardiomyocytes and modest cardiac hypertrophy with functional deterioration [37]. Hence, the expression levels of mitochondria-related proteins were decreased in the DM group to reflect abnormal mitochondrial function. Bax is required for the physiological fusion of mitochondria from elongated mitochondria via the functional activity of outer mitochondrial membrane (OMM) proteins including Mfn2, which inhibit cytochrome c release [38]. The treatment effect of WXKL improved on these proteins.

*I*_Na_ blockers including ranolazine and WXKL can effectively suppress AF [15, 16, 39]. *I*_Na_ blockade of WXKL is atrial-selective in animal coronary perfused atrial preparations at concentrations that cause no effect in the ventricles [13]. Hu et al. suggested that this character of WXKL is due to more negative steady-state inactivation, less negative resting membrane potential, and shorter diastolic intervals in atrial cells [13]. Ranolazine can maintain mitochondrial function and reduce mitochondrial ROS levels in cardiac disorders [40]. The protection of ranolazine against AF development is due to inhibition of late sodium current (*I*_NaL_), reducing intracellular calcium overload [41, 42]. We also found that in DM, MMP of the atrial myocardium is significantly decreased but this was improved by WXKL treatment. These findings are consistent with the preservation of mitochondrial respiration. The effects of WXKL and ranolazine on mitochondria may be similar.

Our research was aimed at clarifying the signaling pathway of WXKL intervention. H_2_O_2_ treatment occurred with the outbreak of ROS, which was related to the change of mitochondria (opening of mitochondrial permeability transition pore and decrease in MMP). H_2_O_2_ can diffuse across the membrane and generate other ROS. Superoxide released into the mitochondrial matrix can react with mitochondrial DNA (mtDNA), and ROS-induced mtDNA damage leads to respiratory complex enzyme dysfunction [43]. H_2_O_2_ delivered to the cytoplasm is involved in a variety of signaling pathways, including mitogen-activated protein kinase (MAPK), c-Jun N-terminal kinase (JNK1), and TNF-*α*/NF-*κ*b [44–46]. Through the activation of these signaling pathways, the mitochondrial dysfunction of atrial fibroblasts led to collagen production and myofibroblast differentiation, decreased cell viability, and increased apoptosis [18]. We demonstrated that WXKL improved mitochondrial function in vitro. Therefore, WXKL can regulate the activation of the signaling pathway induced by H_2_O_2_ to prevent profibrotic cellular activities. Low concentrations of WXKL inhibit *I*_Na_ and suppress acetylcholine-induced AF, while higher concentrations inhibit fast sodium current and prevent ventricular reentrant arrhythmias. It exerts a tonic block on peak *I*_Na_ without significant use dependence and interacts with its inactivated state [13]. WXKL binds to the inactivated state of peak *I*_Na_ and dissociates rapidly. WXKL is speculated to possess antisinus arrhythmia, antiheart failure, and antihypertensive properties [47]. The mechanism of WXKL for mitochondrial function still needs validation in future work.

Several limitations of our study should be noted. Firstly, we did not block the pathway of mitochondrial oxidation *in vitro*, and we could not further clarify that ROS was a sole signaling pathway to improve mitochondrial function by WXKL. OCR of atrial fibroblasts was used as a marker of mitochondrial function. Secondly, we only assessed the effects of WXKL in atrial fibroblasts, and potential effects of WXKL on atrial cardiomyocytes were not investigated. Thirdly, ion channel currents of WXKL intervention were not studied in this study. Moreover, detailed electrophysiological mechanisms of the antiarrhythmic effects of WXKL, such as alterations in autonomic tone, calcium handling, and depolarization and repolarization dynamics, remain to be elucidated. In addition, action morphology will be measured in the presence and absence of WXKL in single atrial myocytes isolated from normal and diabetic hearts. WXKL may have non-DM-related biological effects in control rats. Further studies need to detail all the mechanisms of WXKL on the atrium by regulating the DM-induced mitochondrial oxidative stress.

## 5. Conclusion

WXKL can inhibit oxidative stress and improve mitochondrial function in vitro induced by H_2_O_2_ and in vivo induced by DM, alleviate atrial remodeling, and reduce AF incidence in DM rats.

## Figures and Tables

**Table 1:** Baseline characteristics and serum biochemical and oxidative stress parameters of rats in the three groups.

	Control group (*n* = 8)	DM group (*n* = 8)	DM+WXKL group (*n* = 8)	*P* value
Weight (g)	492.12 ± 22.91	578.00±76.39^∗∗^	566.62 ± 56.40^∗^	0.012
Heart weight ratio (1/1000)	2.53 ± 0.24	3.18±0.44^∗∗^	3.11 ± 0.51^∗^	0.009
Blood glucose (mmol/L)	5.98 ± 0.93	25.76±5.26^∗∗^	25.51±4.93^∗∗^	<0.001
IVS (mm)	1.90 ± 0.21	1.77 ± 0.23	1.75 ± 0.20	0.337
LVPW (mm)	1.93 ± 0.25	2.17 ± 0.33	2.12 ± 0.25	0.213
*E*/*A* ratio	1.52 ± 0.27	1.61 ± 0.63	1.69 ± 0.58	0.823
Insulin (pmol/L)	20.44 ± 5.71	32.65±8.69^∗∗^	31.32±7.25^∗∗^	0.006
hs-CRP (mg/L)	11.90 ± 2.53	17.66±5.19^∗∗^	16.81 ± 3.65^∗^	0.017
SOD (U/mL)	117.00 ± 15.88	99.48 ± 14.78^∗^	114.42 ± 6.55^#^	0.030
MDA (nmol/L)	8.12 ± 1.26	13.82±2.84^∗∗^	9.42 ± 2.05^##^	<0.001
BUN (mmol/L)	7.28 ± 1.30	7.95 ± 1.60	6.81 ± 1.51	0.324
Cr (*μ*mol/L)	57.11 ± 5.73	60.43 ± 6.58	58.44 ± 11.37	0.724
HDL-c (mmol/L)	1.04 ± 0.16	1.20 ± 0.40	1.15 ± 0.29	0.567
LDL-c (mmol/L)	0.54 ± 0.10	0.64 ± 0.14	0.61 ± 0.14	0.275
TC (mmol/L)	1.45 ± 0.57	2.34±0.40^∗∗^	2.36±0.59^∗∗^	0.003
TG (mmol/L)	0.66 ± 0.18	1.01±0.20^∗∗^	0.93 ± 0.26^∗^	0.012

Values are expressed as mean ± SD. DM = diabetes mellitus; WXKL = Wenxin Keli; IVS = interventricular septum; LVPW = left ventricular posterior wall; hs-CRP = high-sensitivity C-reactive protein; SOD = superoxide dismutase; MDA = malondialdehyde; BUN = blood urea nitrogen; Cr = creatinine; HDL-c = high-density lipoprotein cholesterol; LDL-c = low-density lipoprotein cholesterol; TC = cholesterol; TG = triglyceride. ^∗^Compared with the control group, *P* < 0.05. ^∗∗^Compared with the control group, *P* < 0.01. ^#^Compared with the DM group, *P* < 0.05^##^Compared with the DM group, *P* < 0.01.

## Data Availability

The data used to support the findings of this study are available from the corresponding author upon request.

## Supplementary Materials

Supplementary Materials Appendix Figure 1: representative atrial fibrillation episodes induced by right atrium (RA) burst pacing. LA: left atrium; RV: right ventricle. Appendix Figure 2: the representative image of hemodynamic data from a Millar catheter. LVEDP: left ventricular end-diastolic pressure; ECG: electrocardiographic. Appendix Figure 3: all western blot results of transcription factor A (TFAM). Appendix Figure 4: all western blot results of dynamin-related protein 1 (Drp1). Appendix Figure 5: all western blot results of mitofusin 2 (Mfn2). Appendix Figure 6: all western blot results of transforming growth factor-*β* (TGF-*β*). Appendix Figure 7: all western blot results of nuclear factor kappa-b (NF-*κ*b). Appendix Figure 8: all western blot results of *α*-smooth muscle actin (*α*-SMA). Appendix Figure 9: all western blot results of collagen I. Appendix Figure 10: all western blot results of collagen III. Appendix Figure 11: all western blot results of Bcl-2. Appendix Figure 12: all western blot results of Bax.
