# Supplementary material for: Wenxin Keli Regulates Mitochondrial Oxidative Stress and Homeostasis and Improves Atrial Remodeling in Diabetic Rats
Source: Oxid Med Cell Longev. 2020 Feb 13;2020:2468031. doi: 10.1155/2020/2468031 (PMC7040409; doi:10.1155/2020/2468031)

Appendix Figure 1. Representative atrial fibrillation episodes induced by right atrium (RA) burst pacing. LA, left atrium; RV, right ventricle.

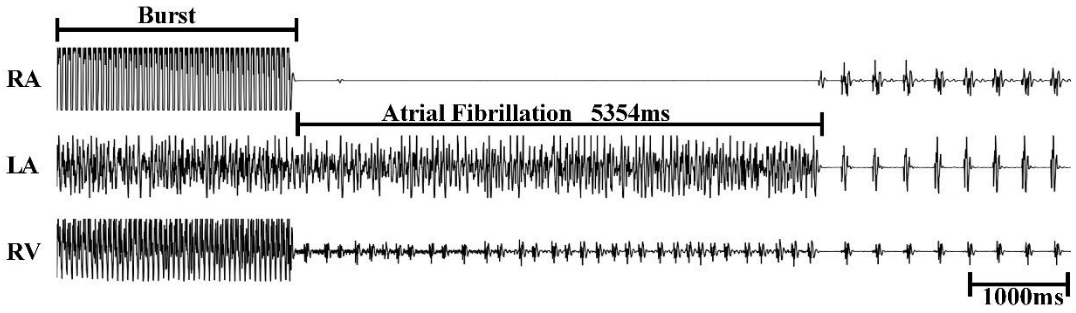

Appendix Figure 2. The representative image of hemodynamic data from Millar catheter. LVEDP, left ventricular end diastolic pressure; ECG, electrocardiographic.

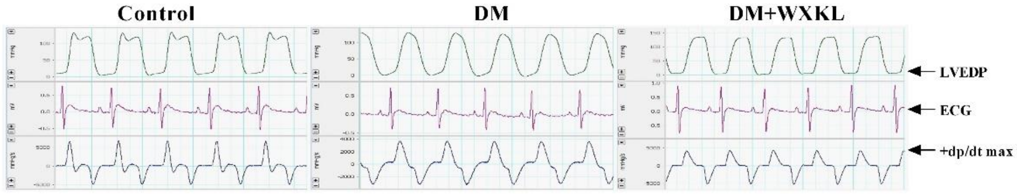

Appendix Figure 3. All western blot results of transcription factor A (TFAM).

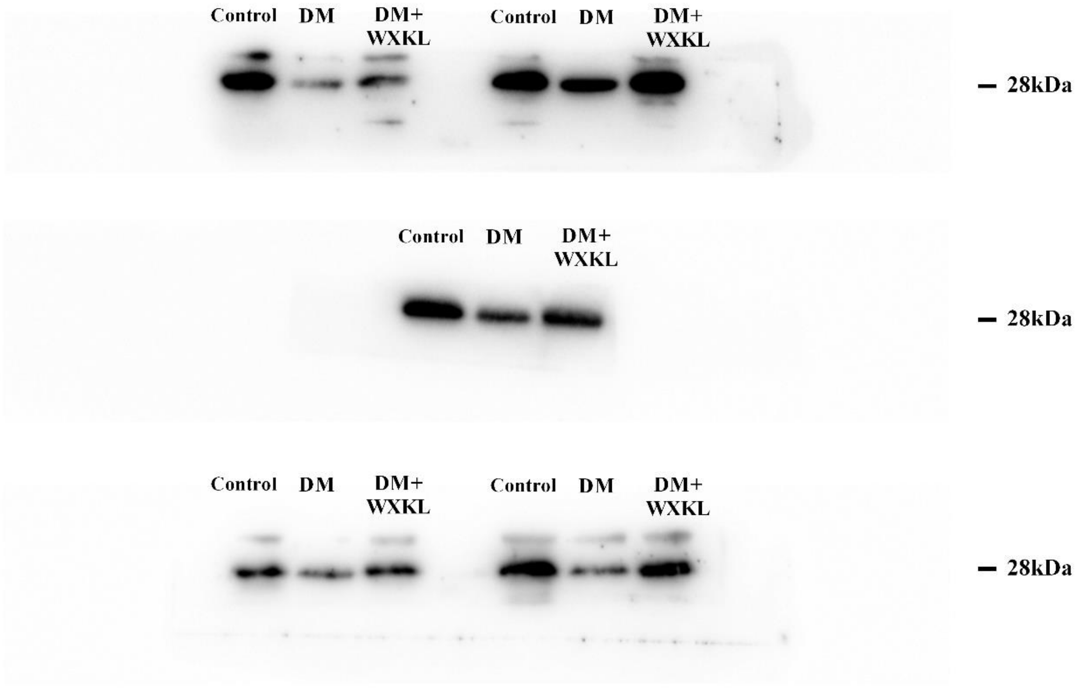

Appendix Figure 4. All western blot results of dynamin-related protein 1 (Drp1).

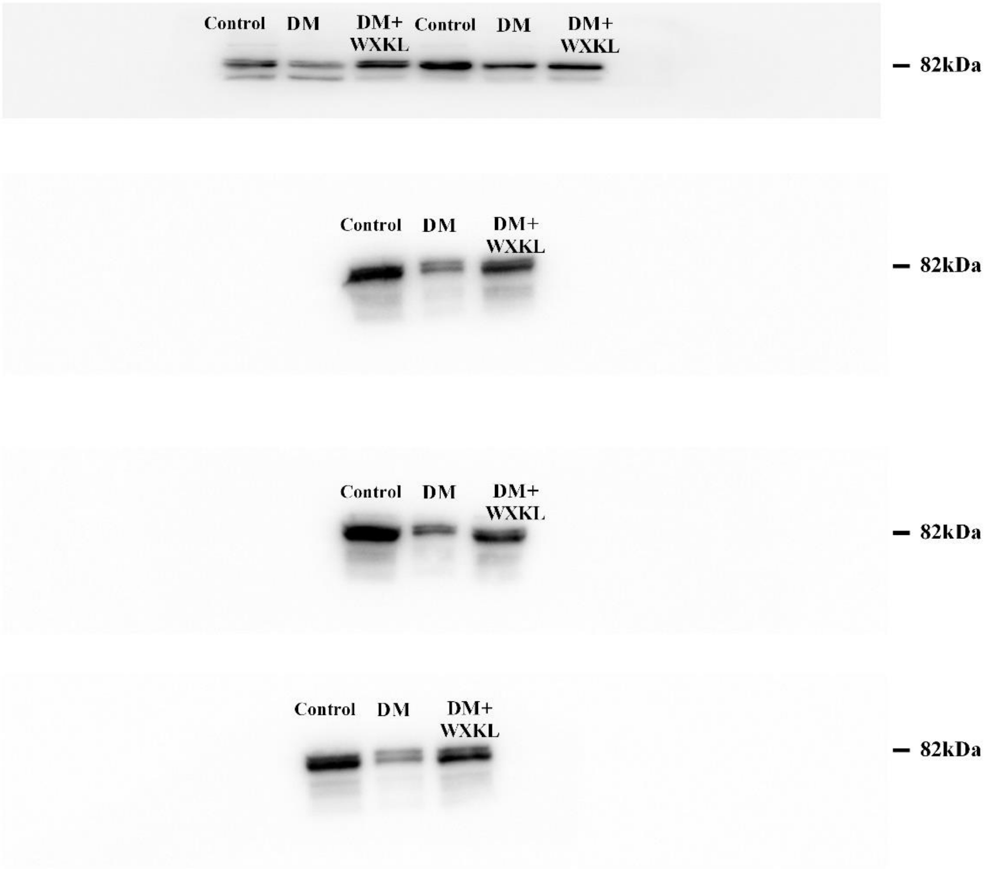

Appendix Figure 5. All western blot results of mitofusin 2 (Mfn2).

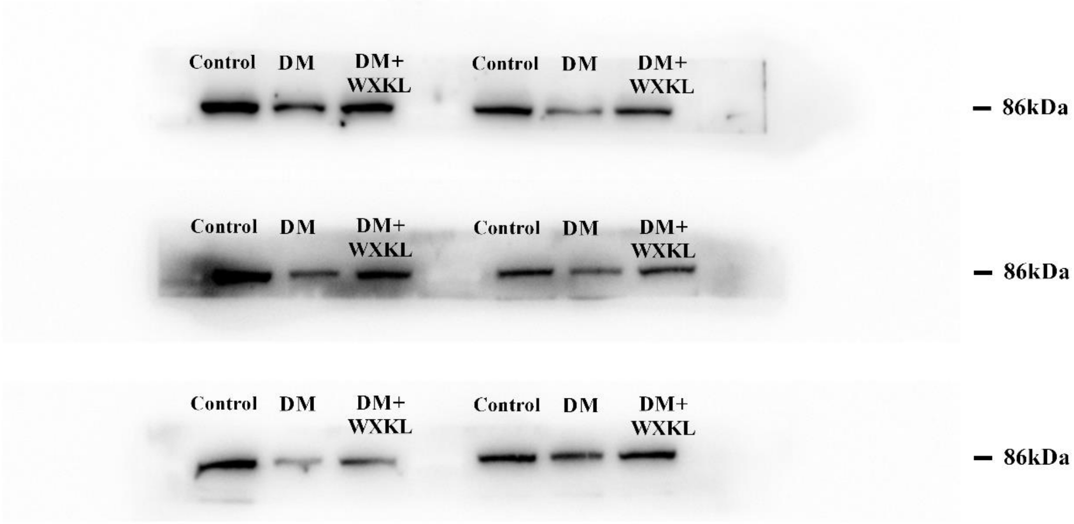

Appendix Figure 6. All western blot results of transforming growth factor-β (TGF-β).

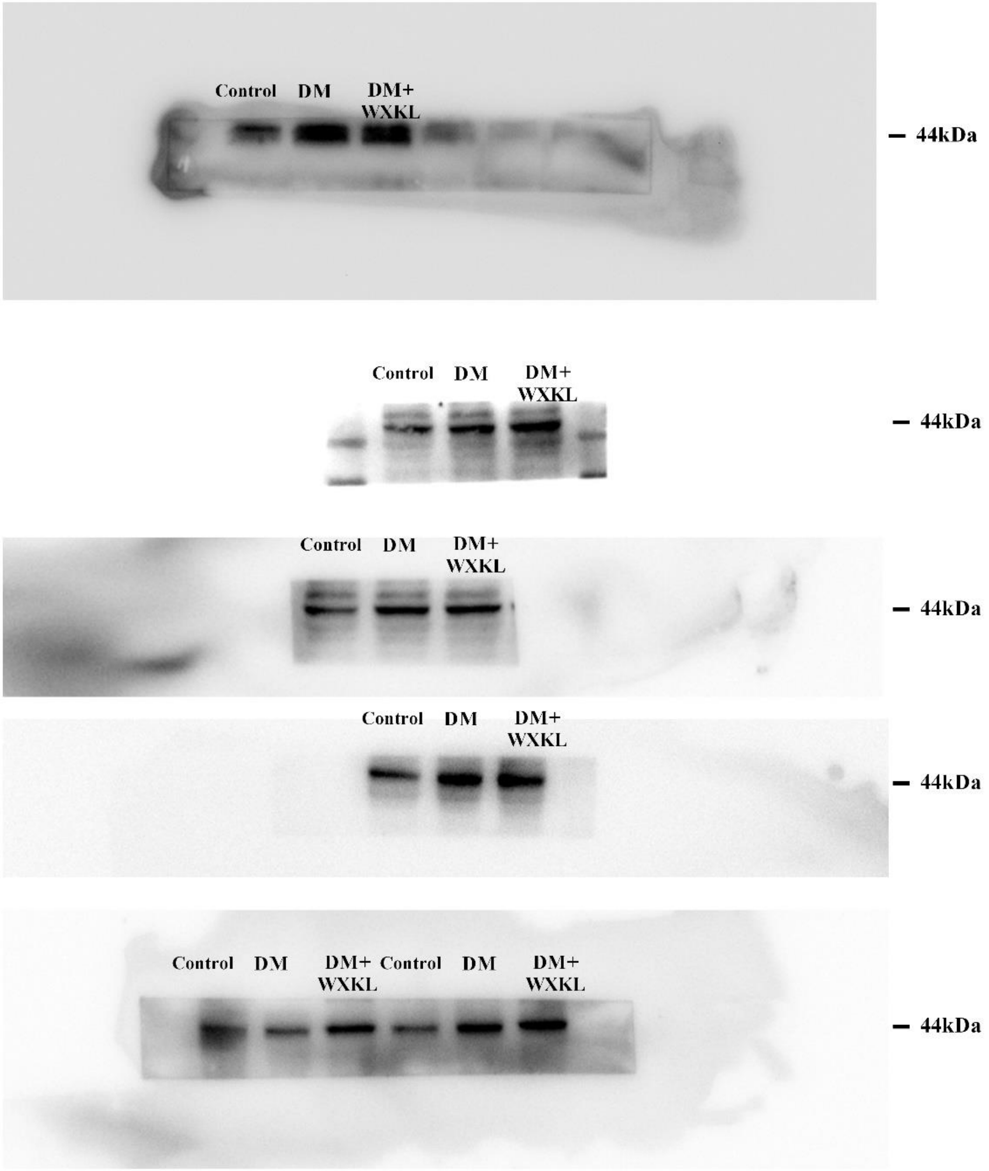

Appendix Figure 7. All western blot results of nuclear factor kappa-b (NF-κb).

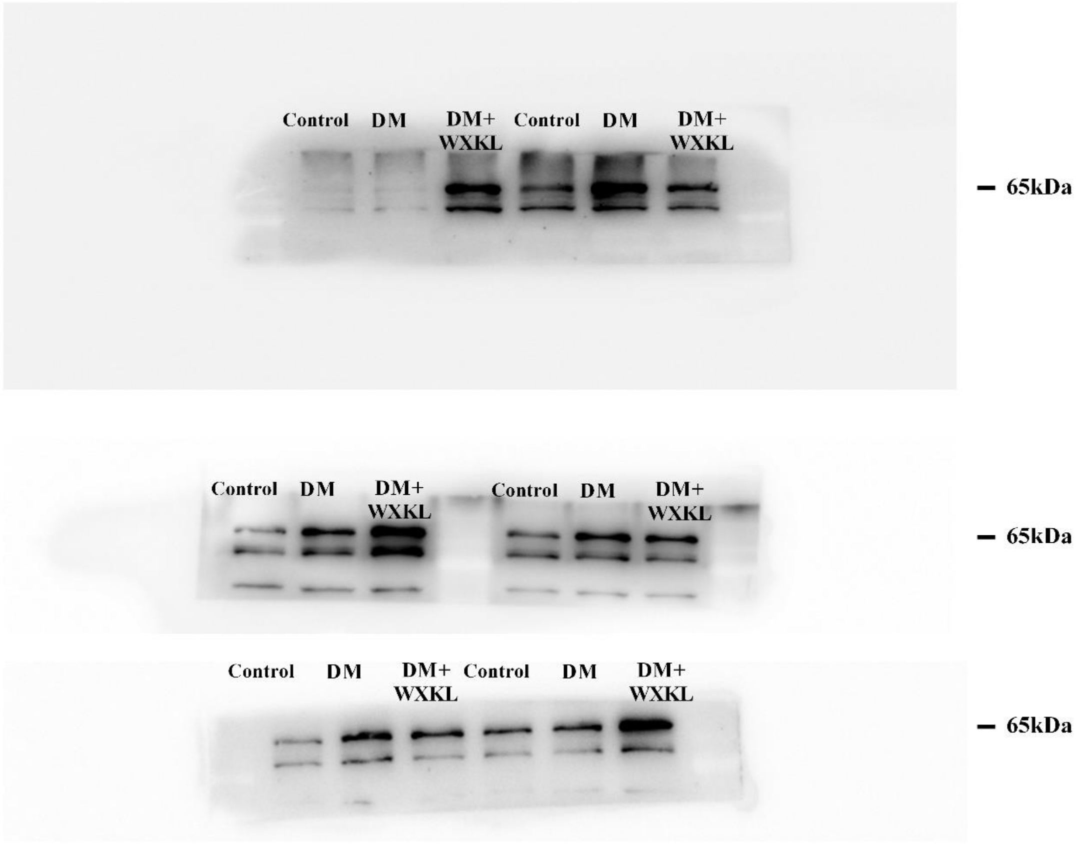

Appendix Figure 8. All western blot results of α smooth muscle Actin (α-SMA).

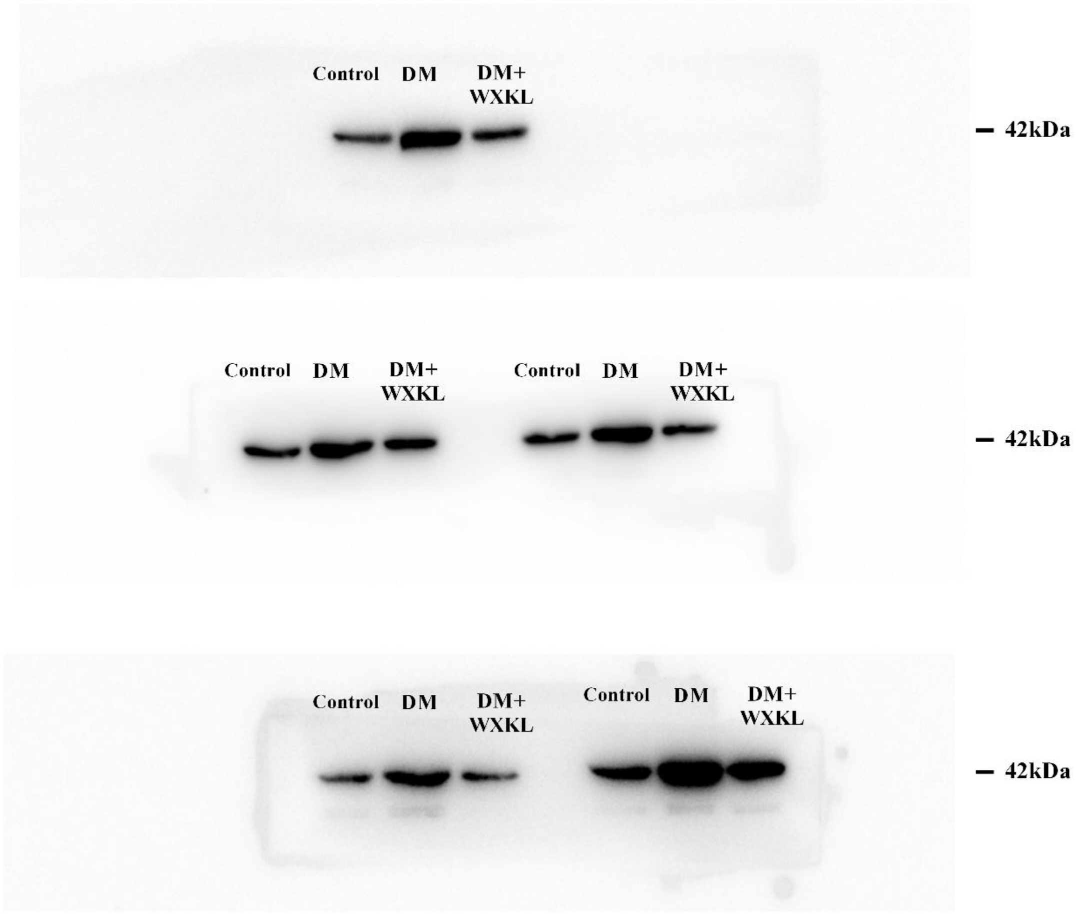

Appendix Figure 9. All western blot results of Collagen I.

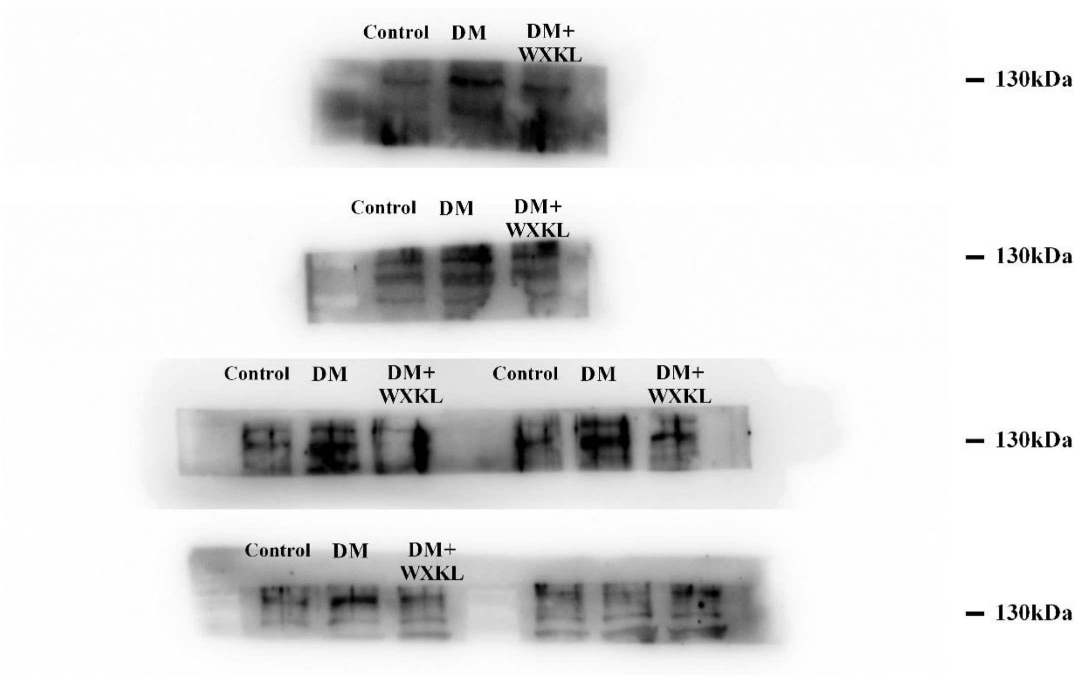

Appendix Figure 10. All western blot results of Collagen III.

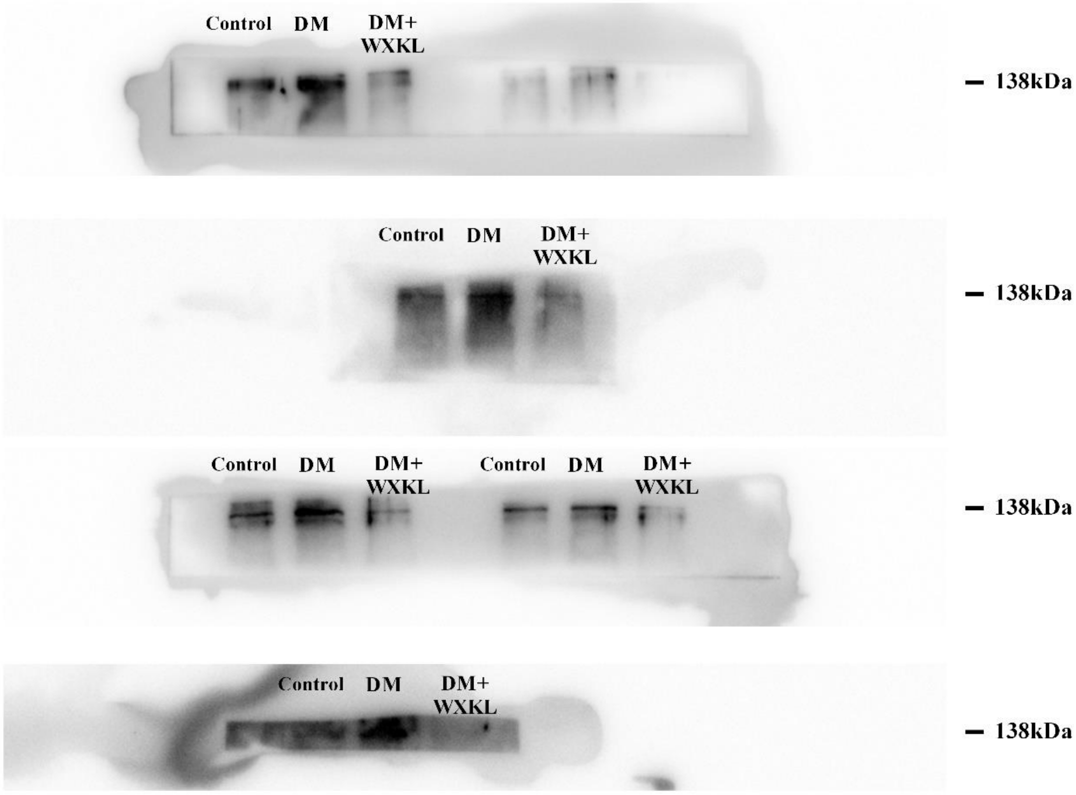

Appendix Figure 11. All western blot results of Bcl-2.

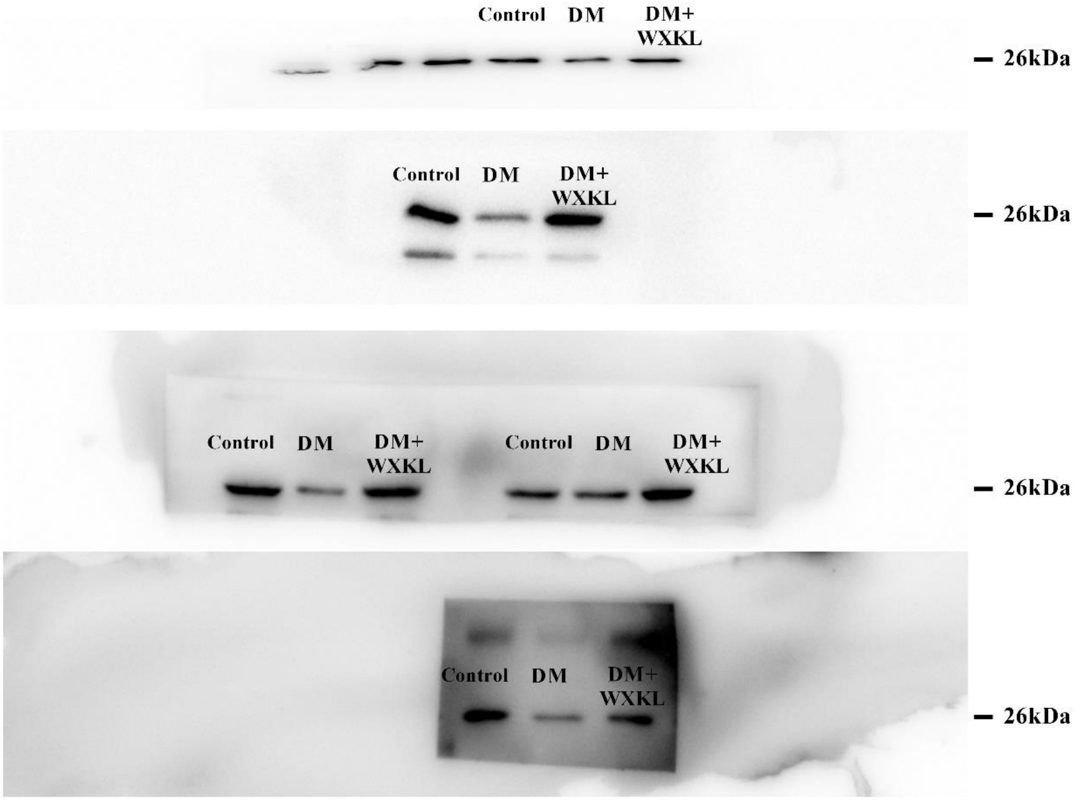

Appendix Figure 12. All western blot results of Bax.

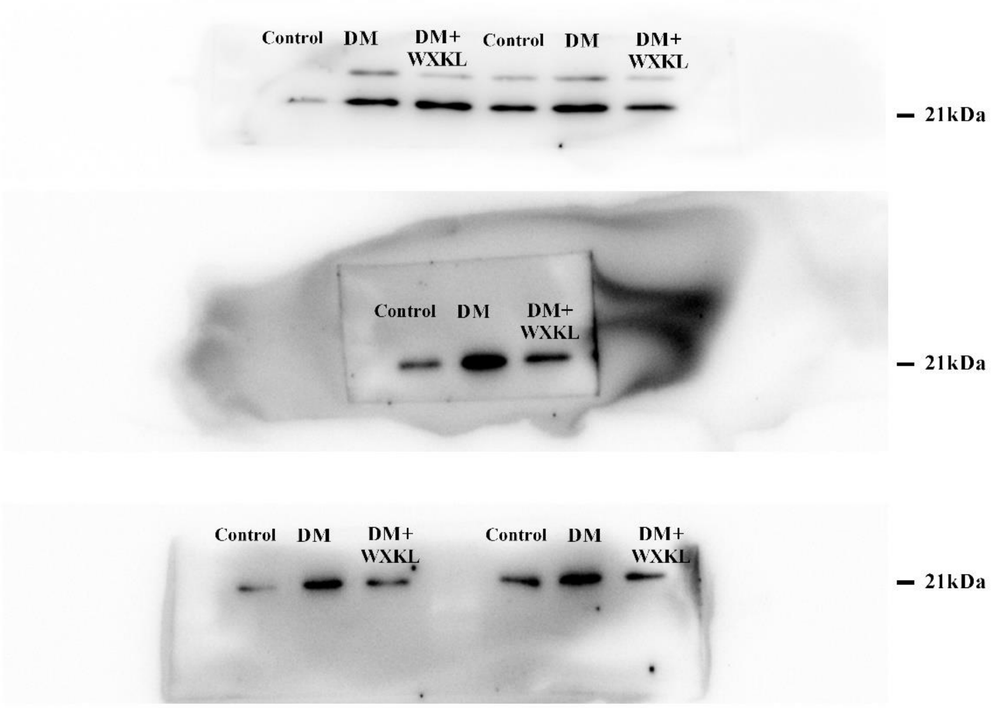

Supplement: Supplementary Materials — Appendix Figure 1: representative atrial fibrillation episodes induced by right atrium (RA) burst pacing. LA: left atrium; RV: right ventricle. Appendix Figure 2: the representative image of hemodynamic data from a Millar catheter. LVEDP: left ventricular end-diastolic pressure; ECG: electrocardiographic. Appendix Figure 3: all western blot results of transcription factor A (TFAM). Appendix Figure 4: all western blot results of dynamin-related protein 1 (Drp1). Appendix Figure 5: all western blot results of mitofusin 2 (Mfn2). Appendix Figure 6: all western blot results of transforming growth factor-β (TGF-β). Appendix Figure 7: all western blot results of nuclear factor kappa-b (NF-κb). Appendix Figure 8: all western blot results of α-smooth muscle actin (α-SMA). Appendix Figure 9: all western blot results of collagen I. Appendix Figure 10: all western blot results of collagen III. Appendix Figure 11: all western blot results of Bcl-2. Appendix Figure 12: all western blot results of Bax. [file 2468031.f1.pdf]
